# Ascorbic acid is associated with favourable hormonal profiles among infertile males

**DOI:** 10.3389/frph.2023.1143579

**Published:** 2023-06-08

**Authors:** Matineh Rastegar Panah, Irtaza Tahir, Bibiana Garcia-Bailo, Kirk Lo, Keith Jarvi, Ahmed El-Sohemy

**Affiliations:** ^1^ El-Sohemy Lab, Department of Nutritional Sciences, Temerty Faculty of Medicine, University of Toronto, Toronto, ON, Canada; ^2^ Temerty Faculty of Medicine, University of Toronto, Toronto, ON, Canada; ^3^ Murray Koffler Urologic Wellness Centre, Division of Urology, Mount Sinai Hospital, University of Toronto, Toronto, ON, Canada

**Keywords:** ascorbic acid, male infertility, hormones, reproduction, sexual health, vitamin C, nutrition, testosterone

## Abstract

**Introduction:**

Infertility affects about 16% of North American couples, with the male factor contributing to ∼30% of cases. Reproductive hormones play an integral role in regulating the reproductive system and consequently, fertility. Oxidative stress reduces testosterone synthesis, and reduction in oxidative stress can improve hormone profiles. Ascorbic acid is a potent antioxidant that accounts for up to 65% of seminal antioxidant activity; however, its effects on reproductive hormones in humans are unknown.

**Methods:**

The objective was to determine the association between serum ascorbic acid concentrations and male reproductive hormones. We conducted a cross-sectional study involving infertile males (*n* = 302) recruited from Mount Sinai Hospital, Toronto. Serum was analyzed for ascorbic acid, luteinizing hormone (LH), follicular stimulating hormone (FSH), total testosterone (TT), prolactin and estradiol. Statistical analyses included Spearman's rank correlations, linear regressions, logistic regressions, simple slope and Johnson-Neyman procedures.

**Results:**

After adjusting for covariates, ascorbic acid was inversely associated with LH (*P* = 0.01). Ascorbic acid was positively associated with TT only among males over the age of 41.6 years (*P *= 0.01).

**Discussion:**

Our findings show that ascorbic acid is associated with higher testosterone levels and improved androgenic status in infertile males, and some of the effects appear to be age dependent.

## 1. Introduction

Infertility, defined as the inability to conceive after 12 months of regular unprotected intercourse, affects nearly 16% of North American couples, with 30% of cases being attributable to male factor infertility and another 20% of cases being attributable to a combination of male and female factor infertility (1). The decline in sperm quality and rise in hypogonadism are contributing to a growing concern regarding male fertility (2, 3). Previous studies have identified a substantial age-independent population-level decline in testosterone concentrations and sperm quality that has been attributed to potential health, environmental and dietary effects (2, 4). Traditionally, primary causes of male infertility were identified as endocrine dysfunctions, physical impairments or genetic polymorphisms (5–7). However, recent research has focused on the potential role of lifestyle factors such as sleep disturbances, body weight, smoking, environmental toxins, and nutrition (8–15). Prudent dietary patterns with high intakes of fruits and vegetables and low intakes of meat, fat, refined sugars, and processed foods have been associated with improved sperm parameters (16–19). A recent review examining the role of nutrition and genetics in male fertility highlighted ascorbic acid, a strong antioxidant, for its potential effects on male reproductive function (20).

Seminal parameters and reproductive hormones are used as surrogate measures of male fecundity and the diagnosis of male factor infertility (21, 22). While most of the research in nutrition and male fertility focuses on seminal parameters, there is a need for further human clinical research exploring the impact of micronutrients on male reproductive hormones that play an integral role in spermatogenesis and subsequently male fertility. The hypothalamic-pituitary-gonadal axis (HPG-axis) is the main signaling pathway responsible for reproductive hormone regulation (23). Gonadotropin releasing hormone (GnRH) secreted by the hypothalamus stimulates gonadotropin release (23). FSH acts predominantly on testicular Sertoli cells to stimulate spermatogenesis and sustain sperm cell maturation vs. luteinizing hormone (LH) acts on Leydig cells to promote intratesticular androgen production, most notably testosterone, necessary for spermatogenesis (23–25). Androgens can be converted to estrogens in the testes and peripheral tissues via aromatase (CYP19) (26). Elevated endogenous estrogen concentrations, especially estradiol, may impair fertility (27). Further, prolactin inhibits the HPG-axis, reducing testosterone synthesis and spermatogenic activity (28). Ultimately, maintaining hormonal homeostasis is fundamental to male reproductive health.

Oxidative stress is important for its role in peroxidative damage induced by reactive oxygen species (ROS), leading to diminished male fertility parameters (14, 29). Testicular oxidative stress is a significant factor in inducing germ cell apoptosis with increased oxidative stress and ROS leading to alterations in spermatogenesis, increased sperm DNA damage and reduced cellular antioxidant activity (13, 30). Moreover, oxidative stress adversely affects testosterone synthesis, leading to impaired hormonal profiles (31, 32). Although there are various factors that can lead to increased oxidative stress, age and adiposity have been identified as two significant contributors. An increase in intracellular ROS coupled with a decrease in antioxidant enzymatic activity leads to an imbalance in testicular redox environment, a physiological manifestation commonly observed with aging (31, 32). Additionally, an increase in adiposity or changes in adiposity distribution with age can contribute to elevated ROS production (13). Thus, as men age, the negative effects of oxidative stress on male fertility parameters become increasingly pronounced. By reducing oxidative stress, it is possible to prevent the detrimental impacts it has on male fertility parameters. Therefore, high concentrations of antioxidants are naturally present in the testes to safeguard against oxidative damage (33).

Antioxidants promote balanced redox environments and aid in repairing ROS-induced cellular damage. Ascorbic acid is an essential water-soluble vitamin that plays a role in hormone production and collagen synthesis and protects against oxidative damage. It is a co-factor in adrenal steroidogenesis and catecholamine biosynthesis in the adrenal cortex and adrenal medulla, respectively (34). Ascorbic acid scavenges free radicals via its strong intracellular and extracellular aqueous-phase antioxidant capacity (35). To maintain adequate levels of ascorbic acid, dietary intake of vitamin C is critical as humans cannot synthesize vitamin C endogenously (36). Nutrient flux and dietary intake of vitamin C directly influence both serum ascorbic acid and in turn seminal ascorbic acid concentrations (37). Older age and smoking are risk factors for ascorbic acid deficiency as age decreases the absorption and utilization of vitamin C, possibly due to reduced expression of sodium ascorbic acid cotransporters, and smoking increases metabolic turnover of vitamin C (36, 38). Moreover, males are at a higher risk of ascorbic acid deficiency in comparison to females due to metabolic differences and lower consumption of ascorbic-rich foods (36, 39, 40). In males, up to ten times higher ascorbic acid concentrations can be present in seminal plasma in comparison to blood plasma (41, 42). The significantly high concentration of ascorbic acid in seminal plasma emphasizes the critical role of ascorbic acid in male reproduction (43). Ascorbic acid contributes to approximately 65% of seminal antioxidant activity (44). Sufficient dietary intake of vitamin C and optimal serum ascorbic acid concentrations have been associated with improved male fertility parameters (41, 45). Several studies have observed an association between higher dietary vitamin C intake and reduction in impaired sperm cellular function, minimization of sperm cell structural flaws, reduced sperm DNA fragmentation and lower DNA damage (33, 46–48). There is ascorbic acid uptake in testicular tissue and the anterior pituitary gland, two sites critical for hormone synthesis. However, despite promising results from animal models assessing the effect of ascorbic acid on reproductive hormones, the relationship between ascorbic acid and reproductive hormones has not been examined in infertile men (49).

## 2. Materials and methods

### 2.1. Study design and participants

Participants (*n* = 832) were recruited from the Murray Koffler Urologic Wellness Centre at Mount Sinai Hospital in Toronto, Canada. The study is a cross-sectional examination of adult males experiencing infertility. Participants were recruited between June 2019 and August 2021. Individuals who were post-operative, had physical impairments leading to infertility, were unable to provide a venous blood sample or semen sample were ineligible for study participation. Individuals with missing data, recent (<6 months) use of fertility related medication, Klinefelter Syndrome, cystic fibrosis, vasectomy or <4 years previous testicular cancer radiation therapy were excluded from the analyses. After all exclusions, the total sample size was 302 males.

### 2.2. Anthropometric measurements and general health information

All participants were required to complete Mount Sinai Hospital's Men's Health Institute's extensive computerized personal health questionnaire. The questionnaire obtained information pertaining to participant's anthropometric data (self-reported weight and height), ethnicity, occupational information, lifestyle and family history (drug use, alcohol use, smoking habits, etc.), urological history, fertility history, previous infertility treatments, andrology data, past medical and surgical history, androgen deficiency and sexual dysfunction questionnaire, mood questionnaire, medication and Sexual Health Inventory for Men (SHIM) and female partner information.

### 2.3. Nutritional and reproductive hormone biochemical measurements

Venous blood was collected and analyzed concurrently for serum ascorbic acid and reproductive hormones at Mount Sinai Hospital's on-site laboratory or LifeLabs medical laboratory services. The serum reproductive hormones analyzed were FSH, LH, total testosterone (TT), prolactin and estradiol. Estradiol levels below 2,533 pg/dl were reported as <2,533 pg/dl by the laboratory.

### 2.4. Statistical analyses

R Studio (Version 1.1.463) was used for analyses. Statistical significance was set at *P* < 0.05. For subject characteristics, simple descriptive analyses reported on count (%) and mean (±SD) for dichotomous and continuous variables, respectively. Differences between tertiles of serum ascorbic acid groups were compared using the Chi-square test for categorical variables and ANOVA for continuous variables. Non-parametric Spearman's rank correlation coefficient analyses was used to assess the strength and direction of the independent monotonic relationship between serum ascorbic acid and reproductive hormones, excluding estradiol. The Shapiro–Wilk test was used to check for normality. For parametric analyses, reproductive hormones were square root transformed to normalize skewed distribution. All models were assessed for normal residual distribution. Initial analyses assessed the linear association between micronutrient concentrations and reproductive hormones as continuous variables using unadjusted and covariate adjusted linear regressions. Additional models were computed with an interaction term for age and serum ascorbic acid to assess effect modification by age in the relationship between serum ascorbic acid and reproductive hormones. Effect modification by BMI was also assessed in a similar manner. Simple slope and FDR corrected Johnson-Neyman interval analyses were completed for the model(s) with statistically significant interactions. Secondary analyses were completed using logistic regressions where serum ascorbic acid was categorized into tertiles and reproductive hormones were dichotomized into clinically significant categories. Clinical status cut-offs were based on reproductive hormones not within their respective normal ranges: elevated FSH (>12.4 IU/L), elevated LH (>7.8 IU/L), low TT (<265 ng/dl), elevated prolactin (>18 ng/ml) and elevated estradiol (>4,000 pg/dl) (50–53). Prolactin was not assessed in logistic regression analyses due to low variability of elevated prolactin levels in the study population. Multivariable linear and logistic regression analyses were adjusted for clinically relevant covariates: age (years), BMI (kg/m^2^), current alcohol consumption (Yes/No/Unspecified), current smoking status (Yes/No), season (meteorological seasons defined as “Winter” = December 1st to February 28th/29th; “Spring” = March 1st to May 31st; “Summer” = June 1st to August 31st; “Fall” = September 1st to November 30th) and ethnicity (Caucasian, African-Canadian, Asian, Indo-Canadian, Middle-Eastern and Unspecified).

## 3. Results

Descriptive characteristics of participants are shown in Table 1. Mean ascorbic acid concentration was 48.3 ± 29.1 µmol/L. Ascorbic acid deficiency (<11 µmol/L) was observed in 6.0% of the study population, while 18.2% had suboptimal ascorbic acid concentrations (11 to 28 µmol/L) and 75.8% had optimal ascorbic acid concentrations (>28 µmol/L) (54). The mean age was 36.6 ± 5.8 years, and the median age was 36 years. The study population had a mean BMI of 28.0 ± 6.3 kg/m^2^, with 29.1% (*n* = 88) in the normal weight category (BMI < 25 kg/m^2^), 48.3% (*n* = 146) in the overweight category (BMI 25–29.9 kg/m^2^), and 22.5% (*n* = 68) in the obese category (BMI ≥ 30 kg/m^2^), with corresponding mean BMIs of 22.9 ± 1.5 kg/m^2^, 27.2 ± 1.3 kg/m^2^, and 36.1 ± 6.8 kg/m^2^, respectively. Participants were pre-dominantly Caucasian (44.4%), followed by Asian (19.2%), unspecified (15.3%), African-Canadian (8.6%), Middle-Eastern (5.6%), Indo-Canadian (4.6%) and Hispanic (2.3%). The majority of participants were non-smokers (85.8%) and non-alcohol consumers (53.6%). Primary hypogonadism, defined as total testosterone concentrations < 265 ng/dl, was present in 30.1% (*n* = 91) of males. ANOVA and chi-square results showed that BMI (*P *= 0.00004), serum TT (*P *= 0.03), TT clinical status (*P* = 0.01) and serum LH (*P* = 0.049) varied across tertiles of serum ascorbic acid.

**Table 1:** Subject characteristics.

Characteristics	*N* (%)	Mean ± SD	Tertile of serum ascorbic acid^a^			*P* ^b^
			Lowest tertile	Mid-tertile	Highest tertile	
**Ascorbic acid biomarkers**						
Count, *n* (%)			106 (35.1)	99 (32.8)	97 (32.1)	
Serum ascorbic acid (µmol/L)		48.3 ± 29.1	21.5 ± 9.2	45.9 ± 6.2	77.8 ± 27.6	
Ascorbic acid status^c^, *n* (%)						
Deficient	18 (6.0)					
Suboptimal	55 (18.2)					
Optimal	229 (75.8)					
**Factors of clinical relevance**						
Age (years), Mean ± SD		36.6 ± 5.8	36.9 ± 6.5	36.2 ± 5.3	36.6 ± 5.5	0.68
BMI (kg/m^2^), mean ± SD		28.0 ± 6.3	30.3 ± 8.0	26.9 ± 4.2	26.8 ± 5.1	0.00004
Current smoker, *n* (%)						0.78
No	259 (85.8)		89 (84.0)	86 (86.9)	84 (86.6)	
Yes	30 (11.5)		17 (16.0)	13 (13.1)	13 (13.4)	
Alcohol consumption, *n* (%)						0.59
No	162 (53.6)		59 (55.7)	57 (57.6)	46 (47.4)	
Yes	134 (44.4)		45 (42.5)	40 (40.5)	49 (50.5)	
Unspecified	6 (2.0)		2 (1.9)	2 (2.0)	2 (2.1)	
Ethnicity, *n* (%)						0.03
Caucasian	134 (44.4)		46 (43.4)	39 (39.4)	49 (50.5)	
Asian	58 (19.2)		21 (19.8)	25 (25.3)	12 (12.4)	
Unspecified	46 (15.3)		19 (17.9)	12 (12.2)	15 (15.5)	
African Canadian	26 (8.6)		7 (6.6)	12 (12.1)	7 (7.2)	
Middle Eastern	17 (5.6)		2 (1.9)	5 (5.1)	10 (10.3)	
Indo-Canadian	15 (4.6)		6 (5.7)	5 (5.1)	3 (3.1)	
Hispanic	7 (2.3)		5 (4.7)	1 (1.0)	1 (1.0)	
Seasonal variation^d^, *n* (%)						0.69
Fall	98 (32.5)		35 (33.0)	29 (29.3)	34 (35.1)	
Winter	90 (29.8)		29 (27.4)	30 (30.3)	31 (32.0)	
Summer	85 (28.1)		34 (32.1)	30 (30.3)	21 (21.6)	
Spring	29 (9.6)		8 (7.5)	10 (10.1)	11 (11.3)	
**Reproductive hormones**						
Serum FSH (IU/L)		9.6 ± 9.4	10.0 ± 10.0	10.5 ± 10.6	8.3 ± 7.0	0.22
Serum LH (IU/L)		7.2 ± 4.8	7.7 ± 5.0	7.6 ± 5.5	6.2 ± 3.4	0.05
Serum TT (ng/dl)		377.8 ± 184.6	340.3 ± 184.6	395.1 ± 201.9	400.9 ± 158.6	0.03
Serum prolactin (ng/ml)		8.8 ± 3.6	8.5 ± 3.4	9.2 ± 4.1	8.5 ± 3.3	0.30
FSH clinical status^e^, *n* (%)						0.65
Normal	229 (75.8)		81 (76.4)	72 (72.7)	76 (78.4)	
Elevated	73 (24.2)		25 (23.6)	27 (27.3)	21 (21.6)	
LH clinical status^e^, *n* (%)						0.08
Normal	202 (66.9)		64 (60.4)	65 (65.7)	73 (75.3)	
Elevated	100 (33.1)		42 (39.6)	34 (34.3)	24 (24.7)	
TT clinical status^e^, *n* (%)						0.01
Normal	211 (69.9)		63 (59.4)	74 (74.7)	74 (76.3)	
Low	91 (30.1)		43 (40.6)	25 (25.3)	23 (23.7)	
Prolactin clinical status^e^, *n* (%)						0.34
Normal	295 (97.7)		104 (99.0)	95 (96.0)	96 (98.0)	
Elevated	7 (2.3)		1 (1.0)	4 (4.0)	2 (2.0)	
Estradiol clinical status^e^, *n* (%)						0.98
Elevated	233 (77.2)		82 (77.4)	76 (76.8)	75 (77.3)	
Normal	69 (22.8)		24 (22.6)	23 (23.2)	22 (22.7)	

^a^ Tertile of serum ascorbic acid cut-offs are ≤35 µmol/L, >35 µmol/L to <56 µmol/L and ≥56 µmol/L for low, mid- and highest tertile, respectively.

^b^ Differences between groups were compared using chi-square for categorical variables and ANOVA for continuous variables.

^c^ Serum ascorbic acid concentration cut-offs are <11 µmol/L, 11 to 28 µmol/L, and >28 µmol/L for deficient, suboptimal and adequate.

^d^ Seasonal variation was determined based on meteorological seasons blood was drawn for analysis, “Winter” = December 1st to February 28th/29th; “Spring” = March 1st to May 31st; “Summer” = June 1st to August 31st; “Fall” = September 1st to November 30th.

^e^ Clinical status cut-offs were based on reproductive hormones not within normal range: elevated FSH (>12.4 IU/L), elevated LH (>7.8 IU/L), low TT (<265 ng/dl), elevated prolactin and elevated estradiol (>4,000 pg/dl).

The results of a non-parametric Spearman's rank correlation coefficient analyses, presented in Table 2, show a statistically significant independent monotonic relationship between serum ascorbic acid and TT (*ρ* = 0.15, *P* = 0.006) There was no significant correlation between ascorbic acid and FSH (*ρ* = −0.05, *P* = 0.42), LH (*ρ* = −0.10, *P* = 0.10) or prolactin (*ρ* = −0.06, *P* = 0.32). Estradiol was not assessed as it was dichotomized into normal (≤4,000 pg/dl) and elevated (>4,000 pg/dl) levels due to lab measurement sensitivity.

**Table 2:** Spearman's rank correlation for the independent association between serum ascorbic acid concentrations and serum reproductive hormone concentrations.

Reproductive hormones	*ρ*	*P*
FSH	−0.05	0.42
LH	−0.10	0.10
TT	0.15	0.006
Prolactin	−0.06	0.32
Estradiol^a^	–	–

^a^ Spearman's rank correlation test not completed for estradiol as estradiol was dichotomized into normal (≤4,000 pg/dl) and elevated (>4,000 pg/dl) level due to lab measurement sensitivity.

The linear relationship between serum ascorbic acid concentrations and square root transformed reproductive hormones was assessed. Prior to covariate adjustments, there was an inverse association between serum ascorbic acid and LH (β = −0.005, *P* = 0.009). After adjustment for covariates, the inverse relationship between ascorbic acid and LH remained significant (β = −0.004, *P* = 0.01). All other univariate and multivariate linear regressions between ascorbic acid and reproductive hormones (FSH, TT, prolactin and estradiol) were not statistically significant. Univariate and multivariate regression results are reported in Table 3.

**Table 3:** Beta-coefficient (±SE) and corresponding *P* for the linear association between serum ascorbic acid concentration (µmol/L) and serum reproductive hormones concentrations.

Reproductive hormones^a^	Unadjusted model β ± SE	Unadjusted *P*	Adjusted model β ± SE^b^	Adjusted *P*	Interaction by age *P*^c^	Interaction by BMI *P*^d^
FSH	−0.003 ± 0.003	0.19	−0.003 ± 0.03	0.14	0.16	0.35
LH	−0.005 ± 0.002	0.009	−0.004 ± 0.002	0.01	0.07	0.12
TT	0.003 ± 0.002	0.07	0.001 ± 0.01	0.36	0.01	0.68
Prolactin	−0.0008 ± 0.001	0.47	−0.0003 ± 0.001	0.79	0.85	0.18
Estradiol^e^	−0.004 ± 0.004	0.42	−0.002 ± 0.005	0.60	0.15	0.97

^a^ Reproductive hormones are square root transformed. The relationship depicted is the association between serum ascorbic acid concentration and square root of reproductive hormones (FSH, LH, TT and prolactin).

^b^ Model adjusted for potential covariates: age, alcohol consumption status, BMI, ethnicity, seasonal variation, and smoking status.

^c^ *P* for covariate adjusted linear regression model assessing the association between ascorbic acid concentration and serum reproductive hormones with effect modification by age (age by ascorbic acid interaction term).

^d^ *P* for covariate adjusted linear regression model assessing the association between ascorbic acid concentration and serum reproductive hormones with effect modification by BMI (BMI by ascorbic acid interaction term).

^e^ Estradiol was assessed categorically, defined as normal (≤4,000 pg/dl) and elevated (>4,000 pg/dl).

**Figure 1:**
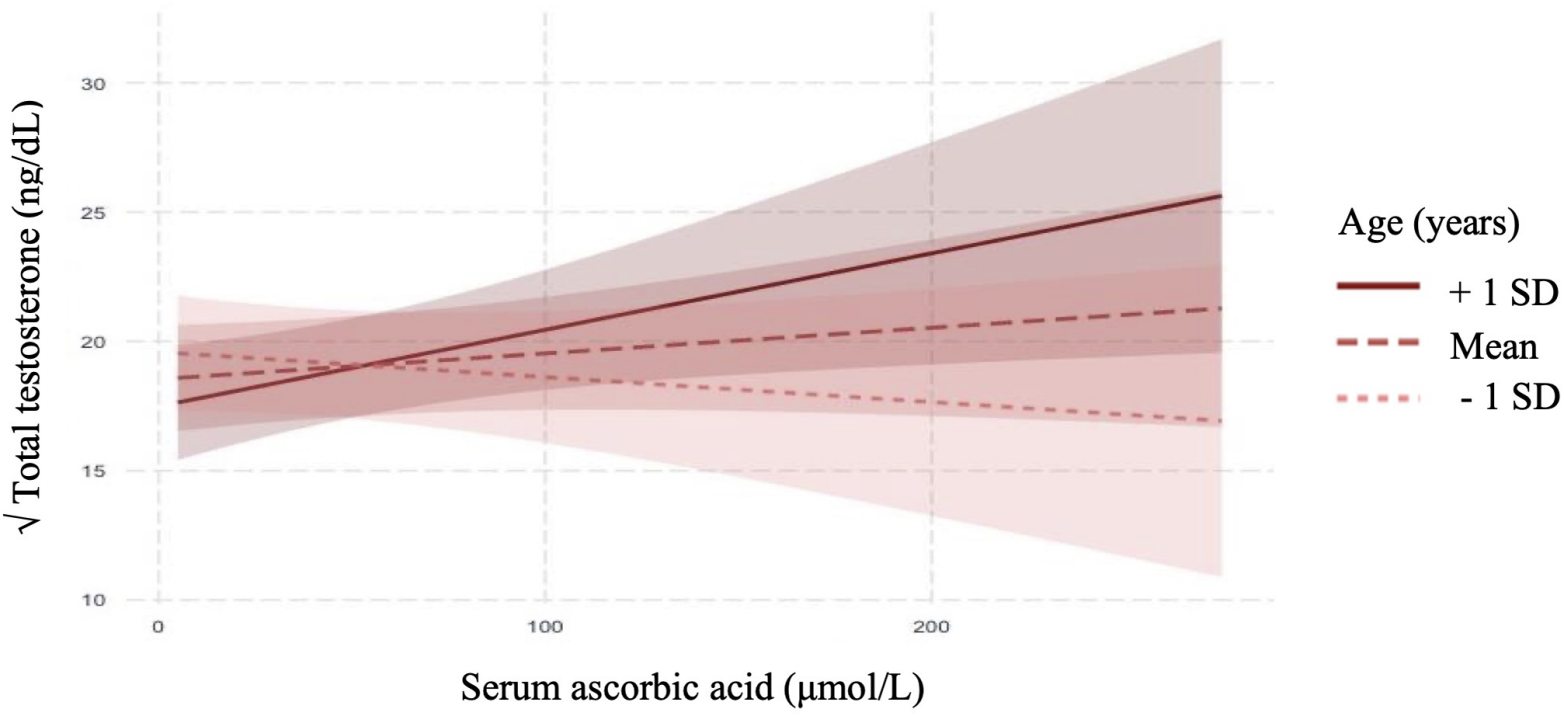
Adjusted relationship between serum ascorbic acid and serum total testosterone modified by age (mean ± SE).

**Figure 2:**
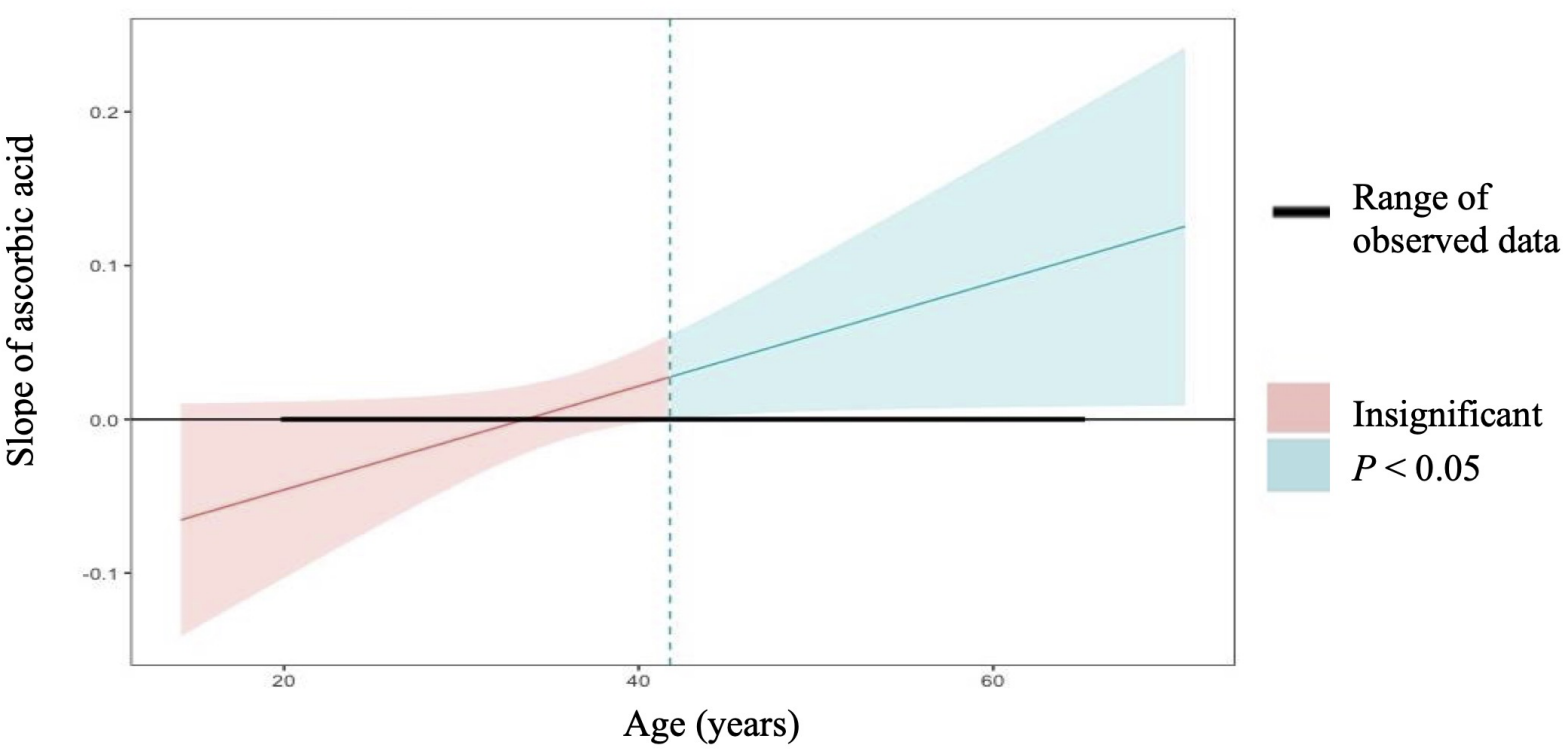
Conditional slope analysis and johnson-neyman interval for the slope of ascorbic acid on total testosterone modified by age^1^. ^1^Johnson-neman interval calculation adjusted for FDR depicts that the slope of ascorbic acid on total testosterone is significant (*P* < 0.05) at all points when age is >41.63 years.

**Figure 3:**
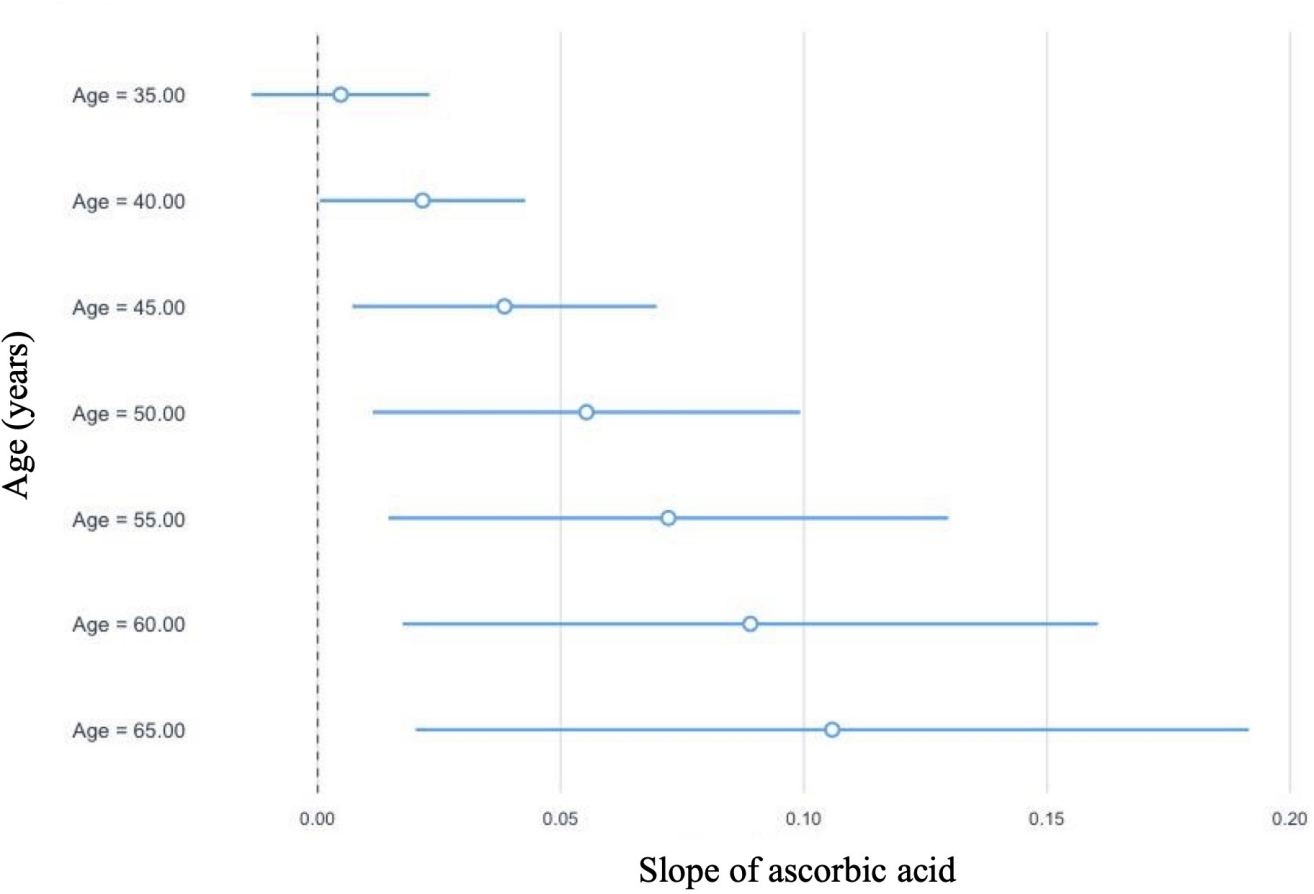
Graphical representation of the conditional slope analysis of ascorbic acid and total testosterone at varying intervals of age.

In linear regression analyses with the inclusion of an effect modifier (age), the interaction between ascorbic acid and age was significant for TT, *P* = 0.01 (Table 3). The effect modification was further investigated by a conditional slope and FDR adjusted Johnson-Neyman interval analysis, which found that the slope of ascorbic acid on testosterone was significant (*P* < 0.05) when age was >41.63 years, as shown in Figures 1, 2. Conditional slope analysis of the slope of ascorbic acid on testosterone at varying intervals of age showed an increase in the slope with increasing age, as shown in Table 4 and Figure 3. Thus, there was a positive age-dependent linear association between ascorbic acid and testosterone. Effect modification by age was not statistically significant for all other reproductive hormone outcomes. In linear regression analyses with inclusion of BMI as an effect modifier, the interaction between ascorbic acid and BMI was not significant for all reproductive hormones (FSH, LH, TT, prolactin and estradiol).

**Table 4:** Conditional slope analysis of slope of ascorbic acid on total testosterone at varying intervals of age.

Age (years)	Slope^a^	*P* ^b^
35	0.00	0.69
40	0.00	0.05
45	0.01	0.01
50	0.01	0.01
55	0.01	0.01
60	0.02	0.01
65	0.02	0.01

^a^ Slope of ascorbic acid on total testosterone.

^b^ FDR corrected *P* for slope.

Logistic regression was used to examine the relationship between tertiles of serum ascorbic acid (reference group = lowest tertile) and clinical status of reproductive hormones (reference group = normal levels of reproductive hormones), as shown in Table 5. Prior to covariate adjustment, individuals in the highest tertile of serum ascorbic acid had lower odds of elevated LH in comparison to individuals in the lowest tertile of serum ascorbic acid (OR = 0.51, 95% CI [0.25, 0.95], *P* = 0.03). However, this relationship did not remain significant after covariate adjustment (OR = 0.58, 95% CI [0.28, 1.24], *P* = 0.10). In the unadjusted analyses, individuals in the highest tertile of serum ascorbic acid had reduced odds of low TT in comparison to individuals in the lowest tertile of serum ascorbic acid (OR = 0.46, 95% CI [0.24, 0.95], *P* = 0.01). However, when the model was adjusted for covariates, the relationship was no longer significant (OR = 0.67, 95% CI [0.34, 1.33], *P* = 0.13). Similarly, in unadjusted analyses, individuals in the middle tertile of serum ascorbic acid had reduced odds of low TT in comparison to individuals in the lowest tertile of serum ascorbic acid (OR = 0.49, 95% CI [0.24, 0.99], *P* = 0.02). However, this was not significant after adjusting for covariates (OR = 0.61, 95% CI [0.29, 1.30], *P* = 0.13). Due to low variability of elevated prolactin concentrations in the study population, prolactin was not assessed in logistic regression analyses. For all other remaining reproductive hormones, odds of experiencing reproductive hormone concentrations not within normal range did not differ by tertile of serum ascorbic acid concentrations.

**Table 5:** ORs and 95% CIs for the association between tertiles of serum ascorbic acid concentration and reproductive hormones not within normal range^a^ using binomial logistic regressions.

	Serum ascorbic mid-tertile^b^				Serum ascorbic acid highest tertile^c^			
Hormone	Unadjusted OR [95% CI]	Unadjusted *P*	Adjusted OR [95% CI]^d^	Adjusted *P*	Unadjusted OR [95% CI]	Unadjusted *P*	Adjusted OR [95% CI]^d^	Adjusted *P*
FSH	1.22 [0.59, 2.12]	0.94	1.12 [0.55, 2.28]	0.50	0.89 [0.46, 1.69]	0.93	0.97 [0.47, 2.00]	0.99
LH	0.73 [0.41, 1.31]	0.55	0.88 [0.34, 1.61]	0.65	0.51 [0.25, 0.95]	0.03	0.58 [0.28, 1.24]	0.10
TT	0.49 [0.24, 0.99]	0.02	0.61 [0.29, 1.30]	0.13	0.46 [0.24, 0.95]	0.01	0.60 [0.28, 1.32]	0.13
Prolactin^e^	–	–	–	–	–	–	–	–
Estradiol	0.90 [0.47, 1.72]	0.94	1.03 [0.50, 2.11]	1.00	1.01 [0.53, 1.93]	1.00	1.03 [0.07, 2.09]	1.00

^a^ Clinical status cut-offs were based on reproductive hormones not within normal range, defined as elevated FSH (>12.4 IU/L), elevated LH (>7.8 IU/L), low TT (<265 ng/dl) and elevated estradiol (>4,000 pg/dl). Serum reproductive hormones within normal range was used as the reference category for all logistic regression analyses.

^b^ ORs compare the odds of experiencing reproductive hormones not within normal range for participants in the mid-tertile of serum ascorbic acid concentrations with participants in the lowest tertile of serum ascorbic acid concentrations.

^c^ ORs compare the odds of experiencing reproductive hormones not within normal range for participants in the highest tertile of serum ascorbic acid concentrations with participants in the lowest tertile of serum ascorbic acid concentrations.

^d^ Model adjusted for potential covariates: age, alcohol consumption status, BMI, ethnicity, seasonal variation, and smoking status.

^e^ Prolactin was not assessed in logistic regression analyses due to low variability of elevated prolactin levels in the study population.

## 4. Discussion

In the present study population, 6.0% of males had deficient serum ascorbic acid concentrations, which is approximately double the rate reported in the nationally representative Canadian Health Measures Survey, reporting less than 3% ascorbic acid deficiency in the general Canadian population (55). Our findings show that higher serum ascorbic acid concentrations are associated with more favourable hormonal profiles in infertile males. Kothari et al. found a positive association between serum ascorbic acid and free testosterone in fertile males, but they did not adjust for any covariates (56). We found that ascorbic acid is inversely associated with LH and the linear relationship with ascorbic acid and TT is age dependent. To our knowledge, these findings are novel, as no studies have examined the association between ascorbic acid and reproductive hormones in infertile males previously. Interestingly, our observation aligns with previous studies using rodents. Okon et al. found that ascorbic acid administration at 200 mg/kg (medium dose group) and 400 mg/kg (high dose group) twice daily for 21 days to albino rats resulted in increased total testosterone concentrations in both the medium- and high-dose group in comparison to control (49). The study also found that LH concentrations decreased in the medium dose group compared to control (49). This has several implications for male fertility, as testosterone is critical in supporting spermatogenesis and LH is responsible for stimulating testosterone production via interstitial cells (31, 32). Further, elevated levels of LH can be indicative of insufficient testosterone production by the testes as sufficient testosterone production negatively feedbacks on the HPG-axis to lower LH (24). While we found no association between serum ascorbic acid and FSH, Okon et al. found that ascorbic acid treatment elevated FSH in albino rats in the high dose ascorbic acid group (49).

Although we observed an inverse linear association between ascorbic acid and LH, the linear association between ascorbic acid and TT was age dependent. Age modified the relationship between ascorbic acid and TT, where the association and strength of the evidence between ascorbic acid and TT strengthened with increasing chronological age. This could be attributed to the potential physiological benefit of ascorbic acid, which is a powerful antioxidant that may have a greater impact on testosterone levels in older males who are more susceptible to oxidative stress. Increased cellular oxidative damage is associated with aging, since chronological age increases intracellular ROS and reduces antioxidant enzymatic activity (31, 32). A study using hyperglycemic rats found that 150 mg of vitamin C by gavage reduced oxidative biomarkers, increased testosterone and LH (57). Additionally, as adiposity distribution changes with age, independent of changes in BMI, it leads to higher systemic inflammation, a risk factor for oxidative stress (58). Ascorbic acid, a strong antioxidant with high intratesticular concentration, could potentially restore oxidative balance in testicular and Leydig cellular redox environments, which could explain our findings that ascorbic acid is associated with improved androgenic status among older males (above 41.63 years of age) who are at highest risk of oxidative stress (31, 32). With the age of first time fathers rising, ascorbic acid may have the potential to be protective against the effects of paternal aging on reproductive health (59).

While the pituitary gland is an important site of ascorbic acid uptake, we found no association between ascorbic acid and prolactin concentrations. Similarly, a previous study in cultured rat pituitary cells found that ascorbic acid does not stimulate or inhibit prolactin release directly (60). However, the study did find that ascorbic acid has a strong potentiation effect in reducing the magnitude of dopamine concentration required for prolactin suppression, which may be due to ascorbic acids' role in protecting against dopamine oxidation (60). Further, we did not find a significant association between ascorbic acid and estradiol clinical status (normal vs. elevated).

Our study has several strengths. To our knowledge, this is the first study to comprehensively evaluate the relationship between serum ascorbic acid and several male reproductive hormones. Covariates of clinical significance were determined *a priori* rather than using a data-driven approach. Moreover, covariates were accounted for in the statistical analyses, and the use of validated nutrient biomarkers precludes the potential self-reporting bias that can occur with dietary intake assessment methods. However, some limitations are present. Due to the cross-sectional nature of the study design, causality and temporality cannot be established. All demographic and anthropometric data were self-reported, increasing risk of self-reporting bias. The study is limited by its sample size of 302 participants and further clinical research with a larger sample size is warranted. Further, we did not have information on ascorbic-rich dietary intake patterns to include as a covariate in our analyses. Future research should focus on enhancing the body of evidence surrounding nutrition and male fertility by understanding the underlying physiological mechanisms at play and generating robust evidence using randomized controlled trials. Growing evidence in the field could have several benefits, including the incorporation of inexpensive, relatively side effect-free antioxidants for male infertility management or to support more successful fertilizations from assistive reproductive technologies.

## Data Availability

The datasets presented in this article are not readily available because data will be made available upon reasonable request. Requests to access the datasets should be directed to a.el.sohemy@utoronto.ca.
